# Embolism recovery strategies and nocturnal water loss across species influenced by biogeographic origin

**DOI:** 10.1002/ece3.5126

**Published:** 2019-04-13

**Authors:** Melanie J. B. Zeppel, William R. L. Anderegg, Henry D. Adams, Patrick Hudson, Alicia Cook, Rizwana Rumman, Derek Eamus, David T. Tissue, Stephen W. Pacala

**Affiliations:** ^1^ Department of Biological Sciences Macquarie University North Ryde New South Wales Australia; ^2^ School of Biological Sciences University of Utah Salt Lake City Utah; ^3^ Department of Plant Biology, Ecology, and Evolution Oklahoma State University Stillwater Oklahoma; ^4^ Department of Biology University of New Mexico Albuquerque New Mexico; ^5^ School of Life Sciences University of Technology Sydney Sydney New South Wales Australia; ^6^ Hawkesbury Institute of the Environment Western Sydney University Richmond New South Wales Australia; ^7^ Department of Ecology and Evolutionary Biology Princeton University Princeton New Jersey

**Keywords:** carbohydrate starvation, drought‐induced mortality, embolism recovery, embolism refilling, hydraulic failure, nocturnal stomatal conductance, nonstructural carbohydrates, xylem embolism

## Abstract

Drought‐induced tree mortality is expected to increase in future climates with the potential for significant consequences to global carbon, water, and energy cycles. Xylem embolism can accumulate to lethal levels during drought, but species that can refill embolized xylem and recover hydraulic function may be able to avoid mortality. Yet the potential controls of embolism recovery, including cross‐biome patterns and plant traits such as nonstructural carbohydrates (NSCs), hydraulic traits, and nocturnal stomatal conductance, are unknown. We exposed eight plant species, originating from mesic (tropical and temperate) and semi‐arid environments, to drought under ambient and elevated CO_2_ levels, and assessed recovery from embolism following rewatering. We found a positive association between xylem recovery and NSCs, and, surprisingly, a positive relationship between xylem recovery and nocturnal stomatal conductance. Arid‐zone species exhibited greater embolism recovery than mesic zone species. Our results indicate that nighttime stomatal conductance often assumed to be a wasteful use of water, may in fact be a key part of plant drought responses, and contribute to drought survival. Findings suggested distinct biome‐specific responses that partially depended on species climate‐of‐origin precipitation or aridity index, which allowed some species to recover from xylem embolism. These findings provide improved understanding required to predict the response of diverse plant communities to drought. Our results provide a framework for predicting future vegetation shifts in response to climate change.

## 1 INTRODUCTION

Increasing drought severity, rising temperature, and vapor pressure deficit (VPD) are contributing to increased drought stress in forests in many regions (Anderegg, Kane, & Anderegg, 2013; IPCC, 2013; Williams et al., 2013). The widespread loss of forests has significant impacts on albedo, soil stability, ecosystem services, catchment and regional water and carbon balances, and global biogeochemical cycles (Bonan, 2008). An improved understanding of the traits and strategies that enable trees to avoid mortality is needed, particularly to improve simulation of drought impacts in vegetation models (Adams et al., 2017; Cobb et al., 2017; Lombardozzi, Zeppel, Fisher, & Tawfik, 2017).

Hydraulic failure results when water loss from transpiration exceeds the capacity of the hydraulic pathway to transport water to the canopy. Subsequently, this increases xylem tension, leading to progressive cavitation and embolism, and ultimately stopping water transport through the xylem (Anderegg, Berry, Smith, et al., 2012; McDowell et al., 2011; Schenk, Espino, Mendez, & McElrone, 2013; Sperry, Hacke, Oren, & Comstock, 2002; Sperry, Meinzer, & McCulloh, 2008). Hydraulic failure is linked with a reduced ability of trees to transport stored carbohydrate reserves, potentially leading to carbon limitation associated with metabolism, osmoregulation, and defense functions (Anderegg et al., 2015). Avoiding embolism and recovery of hydraulic conductivity through repair of embolism are key life history strategies in preventing plant mortality (Anderegg, Berry, & Field, 2012; Sperry & Love, 2015; Sperry et al., 2008). While considerable research has addressed plant characteristics contributing to avoidance of xylem embolism (Hacke, Sperry John, Pockman William, Davis Stephen, & McCulloh Katherine, 2001; Pittermann, Sperry, Hacke, Wheeler, & Sikkema, 2006; Sperry & Hacke, 2004), examination of physiological traits that may contribute to recovery is undertaken relatively infrequently. In addition, the physiological mechanisms of refilling embolism are largely unknown (Klein et al., 2018; Nardini et al., 2017). Further, embolism repair is likely to vary across species and biomes (Brodersen & McElrone, 2013; Schenk, 2014). Thus, understanding the diversity of embolism recovery responses and how recovery might change in elevated CO_2_ concentrations will improve predictions of tree species' vulnerability to forest mortality in future climates.

Recovery from embolism under positive root pressure and across seasons is well established (Brodersen & McElrone, 2013; Christensen‐Dalsgaard & Tyree, 2014; Cun‐Yang, Meinzer, & Guang‐You, 2017). However, debate has occurred over whether recovery from xylem embolism occurs under tension and over short timescales (Brodersen & McElrone, 2013; Cochard & Delzon, 2013; Klein et al., 2018; Nardini et al., 2017). While it has been argued that refilling overnight is uncommon and may be a methodological artifact (Cochard & Delzon, 2013; Wheeler, Huggett, Tofte, Rockwell, & Holbrook, 2013), a review of embolism studies using 46 species across 26 plant families suggested significant evidence of overnight refilling (Brodersen & McElrone, 2013), which has been further supported in recent experiments (Klein et al., 2018; Ogasa, Miki, Murakami, & Yoshikawa, 2013; Trifilò et al., 2014) and in vivo observations (Brodersen, Knipfer, & McElrone, 2018). Thus, overnight refilling following rewatering and subsequent recovery of xylem embolism may occur in some species and some tissues, but its prevalence is largely unknown (Klein et al., 2018). Critically, the traits that influence embolism recovery remain unresolved. A detailed study on six species within one plant family found that cavitation resistance was negatively correlated with embolism recovery overnight after rewatering (Ogasa et al., 2013). Other studies suggest that embolism recovery is increased by changes in osmotic gradients, regulated by soluble sugars and ray parenchyma starch (Nardini, Lo Gullo, & Salleo, 2011; Salleo, Trifilo, Esposito, Nardini, & Lo Gullo, 2009; Secchi, Pagliarani, & Zwieniecki, 2017; Secchi & Zwieniecki, 2011; Trifilò et al., 2017), and thus traits relating to parenchyma and starch storage may be important.

Nocturnal stomatal conductance, a widely observed phenomenon, could be related to embolism recovery (Caird, Richards, & Donovan, 2007; Dawson et al., 2007). Nocturnal water loss has been attributed to numerous factors, including “leaky stomata” with no evolutionary advantage, increased nutrient uptake at night, transport of nutrients and ions, and facilitation of embolism refilling (Caird et al., 2007; Dawson et al., 2007; De Dios et al., 2013). Nocturnal loss of water for no apparent carbon gain contradicts stomatal optimization theory, which suggests that plants maximize carbon gain while minimizing water loss or hydraulic damage (Cowan & Farquhar, 1977; Lombardozzi et al., 2017; Wolf, Anderegg, & Pacala, 2016). However, theory suggests that loss of water from the canopy should lead to further decreased plant water potential, suggesting that nocturnal stomatal conductance should impede xylem recovery (Dawson et al., 2007). Further, elevated CO_2_ has been reported to increase water‐use efficiency, and water loss at night (Zeppel et al., 2012,2011) with studies suggesting possible links with embolism refilling (Zeppel, Lewis, Phillips, & Tissue, 2014). Thus, the evolutionary benefit of nocturnal transpiration and whether it is related to embolism recovery as part of species' drought response strategies is unknown.

We examined a suite of physiological variables and traits during a drought‐rewatering experiment, at ambient and elevated CO_2_, to examine patterns of recovery from embolism and avoidance of mortality in eight tree species representing different ecosystems across a 1,000 mm rainfall gradient. We examined relationships between these three processes—xylem refilling, changes in NSCs, and nocturnal stomatal conductance—and tested whether (a) xylem recovery was affected by increased nocturnal stomatal conductance and changes in NSCs (soluble sugars and starch); (b) higher xylem recovery delayed time to mortality during a subsequent lethal drought; and (c) elevated CO_2_ influenced xylem recovery and time to death.

## 2 MATERIALS AND METHODS

### 2.1 Plant material

Four to six replicate seedlings of *Acacia cultriformis, Acacia iteaphylla, Casuarina cunninghamiana, Eucalyptus camaldulensis, Eucalyptus tereticornis, Eucalyptus sideroxylon, Eucalyptus globulus,* and *Toona australis* seedlings from the Cumberland State Forest Nursery, Sydney Australia, were planted individually in 30‐L pots and grown for 12 months in each of two glasshouses: one had “ambient” CO_2_ conditions [400 ppm], and the second, “elevated” CO_2_ [600 ppm]. Glasshouse temperature and relative humidity followed ambient conditions of Sydney, with mean night and day temperature of 22.5 and 33.5°C, respectively. Mean VPD was 0.5 to 3.2 kPa, at night and day, respectively. Each pot received 100 ml of liquid fertilizer (2.5 g/10 L water = 0.25 g/1 L water; Scotts' fertilizer 20:20:20 N:P:K plus trace elements) and Osmocote Plus Low Phosphorus slow release fertilizer once, upon planting. Soil moisture was measured with a soil moisture Probe CS616 placed to a depth of 30 cm in two replicate pots per species, and logged every 15 min by a Campbell CR1000 data logger, following previously described methods (Zeppel et al., 2012) (data not shown).

### 2.2 Wet, drought, and rewatering treatments

Plants were grown for 12 months, and then, hydraulic and carbohydrate properties including percent loss of conductivity (PLC), P50, pressure–volume curves, and vulnerability curves were measured. Gas exchange and vulnerability curves were measured on ambient CO_2_ plants. Ray parenchyma starch and nonstructural carbohydrates (NSCs) were collected during wet, drought, and rewatered periods. Plant characteristics including leaf area, tree height, and leaf temperature were measured using standard procedures (Palmer et al., 2010; Zolfaghar et al., 2014). Samples were collected during three periods: “Wet,” “Drought,” and “Re‐watered.” During the drought and rewatering treatments, plants received minimal water for 5–18 days until predawn leaf water potentials indicated plants were sufficiently stressed, and then, plants were rewatered to field capacity overnight. Tree size differed at sampling (see Table 1) and was used as a covariate to test for the effect of tree size in statistical analyses. Drought measurements were taken on the last day of the drought (at the peak of drought stress), and rewatered measurements were taken 12 hr after rewatering.

**Table 1:** Hydraulic and plant traits under ambient and elevated CO_2_, including days to death (days) xylem recovery index (XRI, unitless), xylem recovery index as a function of drought (XRI_D_, unitless), stem diameter 30 cm above soil, tree height at time of harvest (cm), leaf temperature (^o^C), specific leaf area (SLA, g/cm), P50 (MPa), *P*_max_ (MPa), turgor loss point (MPa), solute potential at zero turgor, (*π*_0_, MPa), relative water content at zero turgor (%), and modulus of elasticity *ε*(MPa)

	Ambient CO_2_								Elevated CO_2_							
	*Euc ter*, R	*Euc cam*, R	*Aca ite*, R	*Aca cul*, R	*Cas cun*, NR	*Too aus*, NR	*Euc glob*, NR	*Euc sid*, NR	*Euc ter*, R	*Euc cam*, R	*Aca ite*, R	*Aca cul*, R	*Cas cun*, NR	*Too aus*, NR	*Euc glob*, NR	*Euc sid*, NR
Days to death (days)	15.1 (1.5)	13.8 (0.9)	21.9 (7.8)	27.2 (2.4)	21.4 (1.3)	33.6 (1.7)	17.3 (1.1)	10.8 (0.5)	12.3 (0.5)	12.5 (1.1)	20.4 (1.9)	20.5 (2.10)	23.9 (2.1)	35.6 (4.8)	19.0 (2.1)	10.4 (0.4)
XRI (Unitless)	0.70	0.94	0.59	0.97	0.31	0.44	0.72	0.80	0.70	0.70	0.76	1.0	0.47	0.36	0.94	0.83
XRI_D_ (Unitless)	−0.22	0.81	0.25	0.77	−0.79	−1.74	−0.53	0.32	0.61	−0.03	0.62	2.04	−1.66	−0.10	2.99	0.57
Stem diameter (cm)	10.1 (0.5)	10.8 (2.3)	19.4 (3.3)	11.9 (2.3)	10.3 (0.5)	13.8 (1.5)	12.4 (1.2)	13.8 (1.9)	13.8 (0.9)	11.2 (1.8)	22.9 (5.9)	16.5 (3.5)	10.4 (0.4)	16.0 (2.1)	12.8 (0.4)	17.1 (2.3)
Tree height (cm)	92.6 (6.6)	105.1 (6.9)	97.0 (3.8)	117.3 (13.3)	133.0 (7.0)	47.4 (4.9)	132.3 (5.7)	79.5 (12.9)	138.9 (10.6)	112.1 (9.0)	101.8 (6.3)	101.0 (18.5)	134.6 (5.6)	65.0 (6.8)	121.4 (6.6)	90.0 (13.8)
Leaf temp (°C)	30.0 (0.9)	30.7 (1.4)	29.5 (0.8)	27.7 (0.6)	28.9 (0.7)	31.8 (0.7)	31.8 (0.4)	29.8 (0.8)	30.5 (0.4)	31.7 (1.0)	31.4 (0.4)	30.9 (0.7)	30.9 (0.3)	32.5 (0.4)	30.9 (0.5)	31.8 (0.7)
SLA (g cm^3^)	126.8 (18.3)	102.0 (7.4)	97.0 (7.4)	99.7 (6.7)	54.9 (9.4)	234.0 (26.4)	115.4 (13.9)	90.1 (7.0)	102.7 (5.3)	105.1 (6.6)	83.2 (6.4)	88.2 (5.7)	39.3 (2.1)	226.8 (16.1)	146.7 (12.2)	74.4 (4.6)
P50 (MPa)	−6.15 (2.9)	−3.44 (0.7)	−2.96 (0.3)	−3.22 (0.6)	−0.9	−2.35 (0.5)	−5.74 (0.2)	−5.87 (0.4)								
*P*_max_ (MPa)	n/a	−7.19 (1.1)	−5.3 (0.4)	−10.1 (1.5)	n/a	−10.13 (1.0)	−6.82 (0.2)	−15.16 (3.3)								
Turgor loss point (MPa)	−1.7 (0.1)	−1.9 (0.1)	−2.1 (0.1)	−1.5 (0.1)	−2.1 (0.1)	−1.7 (0.2)	−1.2 (0.1)	−1.6 (0.1)								
*π* 0 (MPa)	−1.3 (0.1)	−1.6 (0.1)	−1.4 (0.1)	−1.3 (0.1)	−1.8 (0.1)	−1.3 (0.2)	−0.9 (0.1)	−1.1 (0.1)								
RWC TLP (%)	88.6 (1.2)	83.2 (2.0)	75.3 (3.3)	80.0 (2.2)	88.9 (0.5)	88.1 (0.9)	86.1 (1.1)	90.5 (1.3)								
*ε* (MPa)	12.6 (1.4)	11.2 (1.7)	6.2 (1.0)	8.4 (1.2)	18.5 (1.5)	11.3 (1.9)	6.7 (0.7)	13.0 (1.2)								

Notes

Data represent mean (and standard error in brackets).

NR: nonrecovering species; R: recovering species.

### 2.3 Hydraulic measurement methods

#### 2.3.1 Stomatal conductance

To compare the water use of each species, daytime and nighttime stomatal conductance was measured weekly between 12 noon and 14:30 hr with a steady‐state porometer (AP4, Delta‐T Devices, Cambridge, UK) as the plants grew from July to November 2013 prior to the drought measurement. Nocturnal measurements were conducted between 20:00 hr and 22:30 hr (~2 hr after sunset) 4 weeks prior to drought during October 2013. Measurements were conducted on two to three fully expanded leaves. During the drought treatment, daytime stomatal conductance was measured three times per week in November 2013. Stomatal conductance at night (g_s,n_) was measured on the night of rewatering.

#### 2.3.2 Leaf xylem water potential

Xylem pressure potential was measured on each of two to three leaves of two replicated trees of each species. Measurements were made in the week preceding drought, during drought period, and the day of rewatering at predawn and midday. Measurements were made using a Scholander‐type pressure bomb (Plant Water Status Console, Soil Moisture Equipment Corporation, USA), using previously described methods (Macinnis‐Ng, Zeppel, Williams, & Eamus, 2011). Fully expanded, mature leaves were sampled at the top of the plant.

#### 2.3.3 Hydraulic conductivity and native embolism

**Figure 1:**
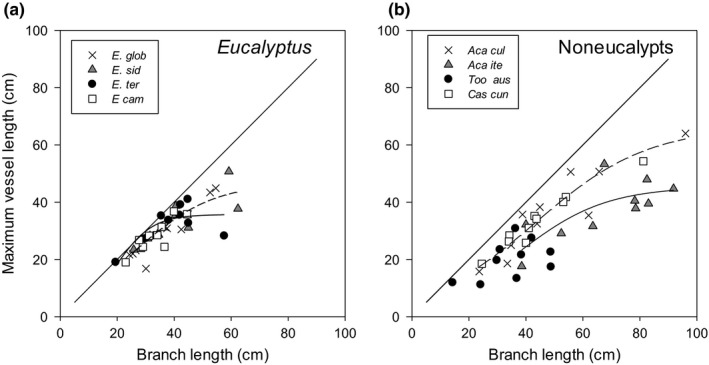
Testing which branch length will be shorter than maximum vessel length. Maximum vessel length and branch length are presented for (a) eucalyptus and (b) noneucalyptus species. Species represent *Eucalyptus globulus,* *Eucalyptus sideroxylon, Eucalyptus tereticornis*, *Eucalyptus camaldulensis*, *Acacia cultriformis, Acacia iteaphylla, Casuarina cunninghamia,* and *Toona australis*(*n* = 8–12). Each data point represents one branch

Native embolism (*K*_h initial_) and maximum hydraulic conductivity (*K*_h max)_ were measured on well‐watered (control), droughted, and then rewatered plants for both CO_2_ treatments, following previously described methods (Choat et al., 2010). Branches were cut underwater and leaves trimmed off flush with the stem and wrapped in parafilm to make one continuous nonleaking stem. Stem ends were shaved with a sharp razor blade, measured in length, and attached to flexible tubing connectors and then to either the tubing of a resistor tubing flow meter. After cutting, branches were left to rehydrate before measurements, to avoid introducing artefacts (Wheeler et al., 2013). To avoid artefacts reported in previous literature, we took the precautions suggested by Wheeler et al. (2013) and Klein et al. (2018). To ensure vessel length was shorter than branch length, we determined maximum vessel length for each species, as a function of branch length (which may be either 2–10 cm less than branch length), using previously reported methods (Klein et al., 2018). This allowed us to determine with confidence that sample branches were longer than maximum vessel length to avoid open vessels (Figure 1). Stems were then flushed with degassed, 0.22 µm filtered 2 mmol potassium chloride solution (KCl) at 100 kPa for 30–40 min until bubbles ceased coming out the end of the stems. Flow rates were then remeasured to calculate the maximum hydraulic conductivity (*K*_h max_) and the initial flow rate divided by *K*_h max_ to determine the native PLC of the stem, (*K*_h initial_/*K*_h max_)*100.

Hydraulic conductivity for *K*_h initial_ and *K*_h__max_ was standardized to sapwood‐specific conductivity (*K*_S_) and leaf‐ specific conductivity (*K*_L_) for well‐watered samples (kg s^−1^ m^−1^ MPa^−1^). Sapwood cross sections were taken from the apex end of the samples, photographed at 10× magnification, and sapwood area measured in Image J.

### 2.4 Pressure–volume measurements

Pressure–volume curves were generated from eight distal leaves or twigs from four well‐watered ambient CO_2_ plants per species, following previously described methods (Eamus, Berryman, & Duff, 1995). For each leaf sample, a branch was cut, then immediately recut under water, and the branch end placed in deionized water, and covered in black polyethylene bags, to rehydrate overnight. Following rehydration, leaves were cut and immediately weighed. Leaf water potential was measured using a pressure chamber (model 1505D, PMS Instruments, Corvallis, OR, USA) and leaf mass immediately reweighed. Leaves were allowed to dry followed by repeated water potential and fresh weight measurements as the leaf‐dried following the bench dehydration method (Turner, 1988) and then oven‐dried at 65°C for 72 hr to obtain dry mass. Pressure–volume (P–V) curves were established by plotting the inverse of leaf water potential (−1/*ψ*) versus relative water content (RWC). Using the dry weight of each sample, the RWC was calculated using the following equation:

(1) $$\text{RWC}=\frac{\left(W_{\text{T}-}W_{\text{D}}\right)-\left(W_{\text{F}-}W_{\text{D}}\right)}{\left(W_{\text{F}-}W_{\text{D}}\right)}*100$$

where *W*_T_ was fully rehydrated leaf weight, *W*_D_ was leaf dry weight, and *W*_F_ was leaf fresh weight. From the P–V curve, several parameters were determined, including RWC, leaf water potential at turgor loss point (*Ψ*_TLP_), RWC at turgor loss point (RWC_TLP_), osmotic potential at full turgor (*π*_100_), modulus of elasticity (*ε*), and saturated water content (Bartlett et al., 2012).

### 2.5 Carbohydrate and water‐use efficiency analysis: nonstructural carbohydrates (NSCs), C13 isotopes

#### 2.5.1 Integrated water‐use efficiency—^13^C isotopes ratios

Well‐watered leaf samples for each species were collected for plants before the drought was imposed and dried at 70°C for 72 hr. Three to four fully expanded, replicate leaves per species were ground and analyzed for the ratio of stable carbon 13 isotopes with Picarro G2101‐*i* CO_2_ analyser and Costech Combustion module (Picarro Inc.) Previously described methods were used (Zolfaghar et al., 2014).

#### 2.5.2 Gas exchange and instantaneous water‐use efficiency

Photosynthesis and g_s_ (mol m^−2^ s^−1^) were measured on four to seven well‐watered mature sun leaves between 9:00 and 15:00 using a Li 6400× (LI COR, Lincoln, NE, USA) 3 weeks prior to drought commencement for baseline maximum gas exchange data following previously described methods (Zeppel et al., 2012). Leaf temperatures were set to 30°C (fluctuated between 28.7 and 31.8°C), flow was set to 500 µmol/s, PARi was set at 1,500 µmol m^−2^ s^−1^, VPD ranged from 1.15 to 2.07 kPa, and CO_2_was set to the level of the glasshouse (400 ppm. or 600 ppm). Instantaneous water‐use efficiency was calculated as the ratio of A/g_s_.

#### 2.5.3 Carbohydrate analysis: nonstructural carbohydrates (NSCs)

To examine NSCs during drought, samples were compared between well‐watered (control), droughted, and rewatered plants. Leaf and stem samples were collected at the time of hydraulic conductance samples and dried at 70°C for 72 hr and then transported to Western Sydney University for analysis. Samples were prepared and analyzed following methods of Tissue and Wright (1995) for soluble sugar and starch concentration. Starch content in the ray parenchyma was measured by staining sections of the sapwood area with lugol solution and examining xylem under a dissecting microscope (Salleo et al., 2009).

### 2.6 Plant characteristics

Leaf area and leaf mass per area were measured using previously described methods (Zeppel et al., 2012). Plant height was measured as standing height prior drought treatment. Specific leaf area (SLA) was calculated from fresh leaf area to dried weight prior drought. Leaf temperature was measured with infrared thermometry (AGRI‐therm III, Everest Interscience Inc.) at midday on one to two leaves per plant.

#### 2.6.1 Wood density

Fresh stem samples were debarked and weighed in a beaker of water using the water displacement method and then dried at 70° C for 72 hr before dry weight was measured. Density was calculated as dry mass/fresh volume as g/ml. Stem diameters were measured 1 cm above soil level or directly above any exposed lignotubers.

### 2.7 Xylem recovery index

The Xylem Recovery Index (XRI), previously described by Ogasa et al. (2013), is based on a function of PLC during two periods: wet and rewatered. Branches were allowed to “rehydrate” in water following cutting to avoid any potential artefacts (Wheeler et al., 2013). XRI is calculated as the ratio of rewatered PLC to the wet PLC (100‐PLC_RW_:100‐PLC_W_). This metric describes PLC while plants are wet and rewatered, however does not account for the PLC a plant reaches during drought. Therefore, we used a modified metric quantified from PLC at three consecutive points, wet, drought, and rewatered, called “XRI‐drought” (XRI_D_), which accounts for embolism recovery from drought with rewatering relative to embolism that occurred during drought (Equation (2)). Positive values mean some level of recovery, and negative XRI_D_ values mean that the PLC during drought was lower than PLC after rewatering, indicating the opposite of embolism recovery. Changes in PLC were used as a proxy to test hydraulic failure along the soil–root–stem pathway following previously established methods (Brodersen & McElrone, 2013; Ogasa et al., 2013; Trifilò et al., 2015).

We quantified xylem recovery using a modified version of a previously published XRI (Ogasa et al., 2013), termed here XRI_D._The XRI was modified to take into account the level of drought that the plant has recovered from, and was calculated using Equation (2). Xylem recovery was calculated from the PLC during three consecutive periods, “wet,” “drought,” and “rewatered” (see methods), using the following equation:

(2) $${\text{XRI}}_{\text{D}}=\frac{{\text{PLC}}_{\text{D}}-{\text{PLC}}_{\text{RW}}}{{\text{PLC}}_{\text{D}}-{\text{PLC}}_{\text{W}}}$$

where PLC_D_, PLCR_W,_ and PLC_W_ are percent loss of branch hydraulic conductivity during drought, wet, and rewatered periods, respectively. The numerator in Equation (2) is xylem embolism recovery with rewatering following drought, while the denominator is the sensitivity of embolism to drought from initial wet conditions. Thus, XRI_D_ quantifies recovery from drought relative to sensitivity to drought (i.e., embolism that occurred during drought).

#### 2.7.1 Time to mortality

Following the rewatering treatment, water was subsequently withheld from plants entirely to determine time to mortality, defined as when the complete canopy was brown with dried leaves. Plants were rewatered a second time (3L) 74 days after the beginning of the second drought to confirm mortality, and no plants were recovered.

### 2.8 Statistical analysis

We used two‐way ANOVA to compare interactions and effects of CO_2_ and species using SPSS v12.0 (2013, Armonk, NY). Data were tested for normality and equal variance and transformed where necessary. To test for the effect of tree size on the independent variable, we used tree size as a covariate, using SPSS v12.0 (2013, Armonk, NY). Vulnerability curves, P50, and P_e_ values were calculated using R statistical software (R Core Development Team, 2010).

## 3 RESULTS

### 3.1 Water relations and days till death

**Figure 2:**
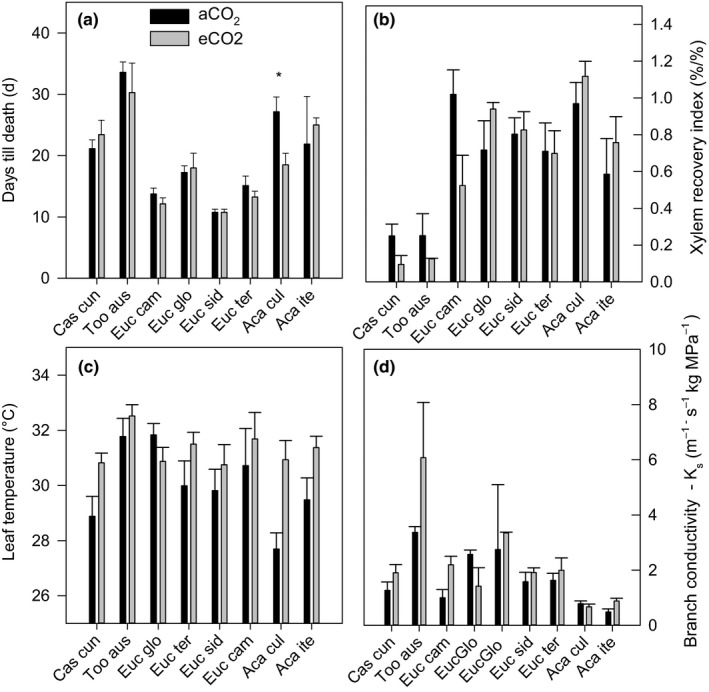
Variability across and within species for (a) days till death, (b) Xylem Recovery Index, (c) leaf temperature, and (d) branch conductivity, for ambient and elevated CO_2_. Species are *Casuarina cunninghamia, Toona australis, Eucalyptus globulus,* *Eucalyptus camaldulensis*, *Eucalyptus tereticornis*, *Eucalyptus sideroxylon Acacia iteaphylla,*and *Acacia cultriformis* (*n* = 8–12). Data represent mean and standard error bars. ANOVA results for species, and CO_2_ and their interactions are shown

**Figure 3:**
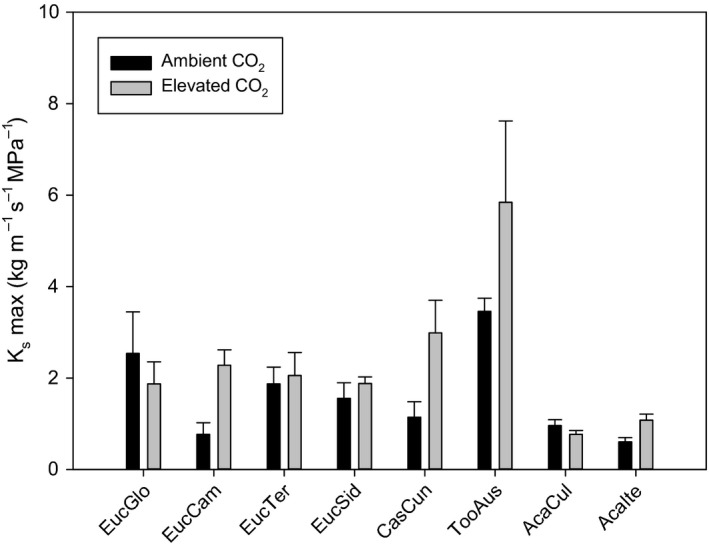
Maximum branch conductivity for all species under elevated and ambient CO_2_. Species are *Eucalyptus globulus,* *Eucalyptus camaldulensis, Eucalyptus tereticornis*, *Eucalyptus sideroxylon, Casuarina cunninghamia, Toona australis,* *Acacia cultriformis,*and *Acacia iteaphylla*(*n* = 8–12). Data represent mean and standard error bars. ANOVA results for species, and CO_2_ and their interactions are shown

There was significant variation across species for branch conductivity (*p* < 0.001), XRI (*p* < 0.01), and days till death (*p* < 0.01; Figure 2). Maximum branch conductivity varied across species, and the variation within species was generally smaller than variation across species (Figure 3).

**Figure 4:**
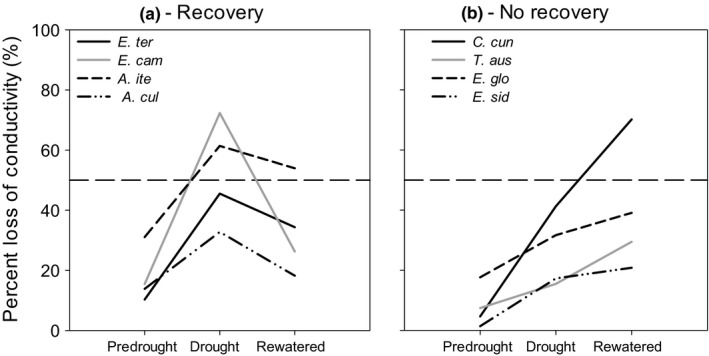
Percent loss of conductivity (PLC) across the course of drought and rewatering, showing predrought (wet), drought, and following rewatering. Panels show (a) four species with positive XRI_D_, where PLC after rewatering recovers back to PLC values lower than those observed during drought, and (b) four species with negative XRI_D_ where PLC continues to increase after rewatering_._The dotted line shows P50, 50% loss of conductivity. Recovery class (R) species are *Eucalyptus tereticornis*, *Eucalyptus camaldulensis*, *Acacia iteaphylla, and Acacia cultriformis*, while No Recovery class species (NR) are *Casuarina cunninghamia, Toona australis, Eucalyptus globulus,* and *Eucalyptus sideroxylon*(*n* = 8–12)

We observed two distinct embolism recovery responses. Four species exhibited strong xylem recovery, where PLC after rewatering was low compared to PLC during drought (hereafter classified as Recovery species, “R”; Figure 4a). The other four species did not exhibit xylem recovery: That is, PLC was higher after rewatering compared with drought (hereafter classified as No Recovery species “NR”), with negative XRI_D_ (Figure 4b). *Eucalyptus tereticornis*, *E. camaldulensis, A. iteaphylla,* and *A*. *cultriformis* were in the class Recovery, and *Casuarina cunninghamia*, *T. australis*, *E. globulus*, and *E. sideroxylon* were in the class No Recovery. Two features in this result are noteworthy. First, the mean (53%) and range (33%–72%) percentage loss of conductivity (PLC) during drought of the four class R species, which exhibited recovery, were larger than the mean (26%) and range (15%–41%) for the four species that did not exhibit recovery. Therefore, we conclude that the lack of xylem embolism recovery in NR species could to be attributed to NR species reaching excessively high PLC relative to R species. This potential explanation can be excluded because the mean and range of PLC of NR species were lower than for R species. Second, 75% of R species were from the arid sites, while 75% of NR species were from the wetter sites (see below).

High xylem recovery rates occur when the PLC is high during drought and PLC is low after rewatering (Figure 4a). In contrast, negative xylem recovery is defined to have occurred when PLC after rewatering is higher than PLC during drought, that is, loss of conductivity continues even after rewatering (Figure 4b). The absence of xylem recovery following rewatering may be caused by a sufficiently large hydraulic failure or deterioration to prevent effective recovery. This hydraulic failure, even after rewatering, may result from a lack of hydraulic connectivity along the soil–root–stem pathway.

**Figure 5:**
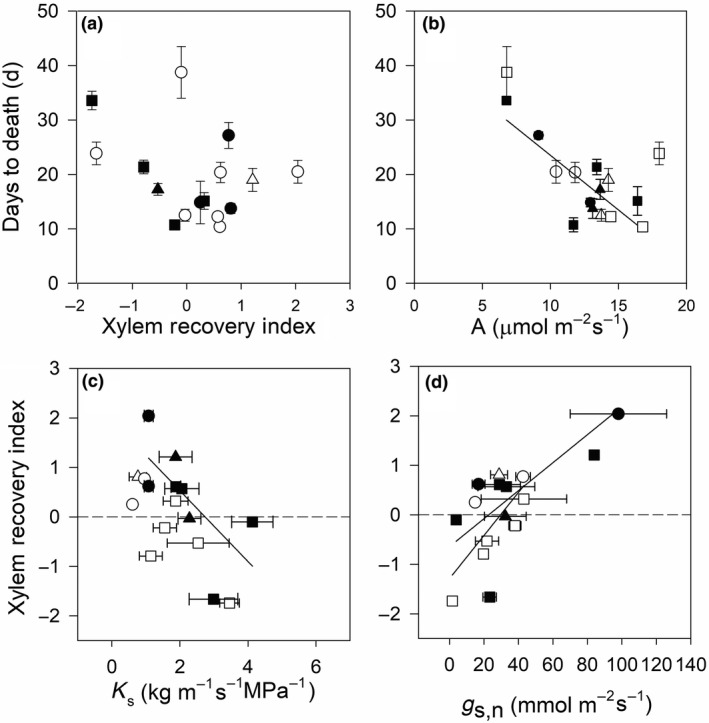
The relationship between (a) days to death and Xylem Recovery Index (XRI_D_) (aCO_2_ *p* = 0.12; eCO_2_ *p* = 0.36) and (b) days to death (days) and photosynthesis (μmol m^−2^ s^−1^, aCO_2_ *p* = 0.012; eCO_2_ *p* = 0.034). The relationship between (c) Xylem Recovery Index (XRI_D_) and sapwood area‐specific branch conductivity and (d) Xylem Recovery Index and stomatal conductance at night. Data points show ambient CO_2_ (closed symbols) and elevated CO_2_ (open symbols) and triangles represent semi‐arid species, circles represent tropical, and squares represent temperate ecosystem species showing the mean and SE of each species (*n* = 4–10). Regressions are presented where relationships are significant. Significance level of relationships is as follows (c) sapwood area‐specific branch conductivity (*K*_s_, kg s^−1^ m^−1^ MPa^−1^; aCO_2_ *p* = 0.01, *R* ^2^ = 0.58; eCO_2_ *p* = 0.27, *R* ^2^ = 0.06) and (d) stomatal conductance at night (g_s,n_, mmol m^−2^ s^−1^; aCO_2_ *p* = 0.01, *R* ^2^ = 0.66; eCO_2_ *p* = 0.01 *R* ^2^ = 0.72). One set of plants is defined by water availability (semi‐arid), and the other two are defined by temperature (tropical vs. temperate)

Stomatal conductance during the day varied from 140 to 426 mmol m^−2^ s^−1^ under ambient CO_2_and 57 to 269 mmol m^−2^ s^−1^ under elevated CO_2_, respectively (Table 2; Figure 5). The ratio of stomatal conductance during the night to day (g_s,n_: g_s,d_) ranged from 4.1% to 26.3% for ambient and 7.2% to 50.2% for elevated CO_2_(Table 2). The species with highest ratio of g_s,n_:g_s:d_ were from arid regions, suggesting a role of nocturnal water loss in plants from water‐limited regions.

**Table 2:** Leaf scale hydraulic traits under ambient and elevated CO_2_

Leaf scale hydraulic traits	Ambient CO_2_								Elevated CO_2_							
	*Euc ter*, R	*Euc cam*, R	*Aca ite*, R	*Aca cul*, R	*Cas cun*, NR	*Too aus*, NR	*Euc glob*, NR	*Euc sid*, NR	*Euc ter*, R	*Euc cam*, R	*Aca ite*, R	*Aca cul*, R	*Cas cun*, NR	*Too aus*, NR	*Euc glob*, NR	*Euc sid*, NR
g_s,d_ (mmol m^−2^ s^−1^)	426 (61)	304 (77)	141 (18)	294 (80)	370 (51)	312 (82)	442 (168)	140 (71)	166 (3)	195 (53)	141 (18)	224 (52)	181 (26)	269 (137)	132 (55)	57 (18)
g_s,n_(mmol m^−2^ s^−1^)	43.16 (24.8)	28.8 (4.9)	14.9 (1.9)	42.0 (4.3)	19.7 (1.2)	1.7 (0.3)	21.8 (6.8)	38.0 (3.5)	33.0 (16.5)	32.4 (12.1)	16.9 (3.7)	98.6 (28.4	23.4 (3.9)	3.9 (2.3)	84.0 ()	29.0 (12.1)
g_s,n_:g_s,d_(%)	12.5	9.5	5.6	26.3	10.9	4.1	6.1	20.4	20.0	15.7	11.3	36.0	27.0	7.2	40.1	50.2
E_t_(mmol m^−2^ s^−1^)	5.2	2.9	1.9	3.3	3.0	1.5	2.4	2.4	1.7	2.0	1.9	2.4	3.0	1.4	1.8	2.1
Critical Leaf water potential (MPa)	−6.3 (07)	−6.3 (3.7)	−9.95 (0.1)	−2.8 (0.8)	−4.2 (0.2)	−2.9 (0.04)	−3.9 (0.1)	−4.3 (1.2)	−5.6 (1.8)	−3.6 (1.2)	−5.8 (05)	−2.2 (0.5)	−3.4 (1.0)	−2.1 (0.2)	−3.3 (0.8)	−7.7 (2.3)
A_sat_(μmol m^−2^ s^−1^)	19.2	13.4	11.2	9.4	14.4	7.6	14.5	15.4	14.8	14.6	15.8	9.2	18.2	7.9	13.6	13.6
Wood density (g cm^3^)	0.4 (0.02)	0.5 (0.09)	0.5 (0.01)	0.5 (0.02)	0.5 (0.05)	0.3 (0.02)	0.6 (0.12)	0.6 (0.08)	0.50 (0.03)	0.54 (0.09)	0.58 (0.03)	0.42 (0.03)	0.47 (0.08)	0.40 (0.04)	0.41 (0.11)	0.49 (0.04)
WUEi (μmol/mol)	52.3 (3.1)	64.8 (3.1)	67.0 (8.8)	65.89 (5.6)	51.1 (9.1)	71.6 (3.1)	71.6 (9.1)	71.6 (3.1)								
A/g_s_ WUE	56.0 (18.7)	67.1 (12.7)	99.4 (10.5)	49.9 (10.8)	72.2 (10.3)	70.6 (6.4)	86.0 (15.0)	89.6 (7.9)	174.0 (33.4)	152.2 (21.2)	134.9 (37.2)	49.7 (6.9)	82.7 (6.2)	111.5 (26.3)	119.6 (16.1)	139.0 (28.1)

Notes

Hydraulic traits including stomatal conductance at day, g_s,d,_and night, g_s,n_ (mmol m^−2^ s^−1^), the ratio of stomatal conductance at night:day (%), leaf level transpiration, E_t_ (mmol m^−2^ s^−1^), critical leaf water potential, or water potential at wilting, photosynthesis at saturated light (mmol m^−2^ s^−1^), wood density, integrated water‐use efficiency (WUEi, from ^13^C isotopes), and instantaneous water‐use efficiency (WUE, A/g_s_ from gas exchange). Data represent mean (and standard error in brackets).

### 3.2 Xylem recovery and carbohydrates

**Figure 6:**
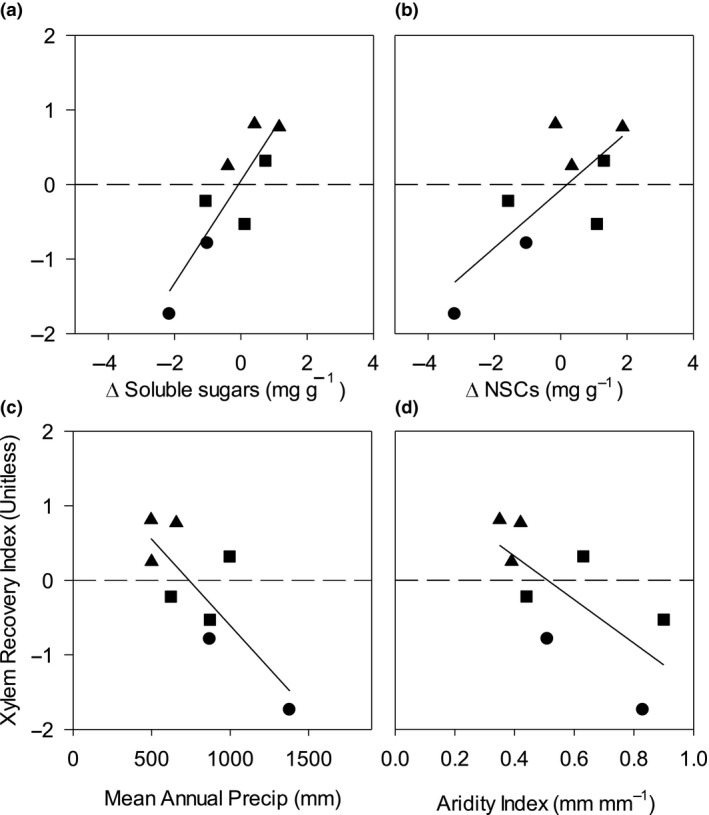
Relationships between Xylem Recovery Index (XRI_D)_and (a) overnight changes in soluble sugars (mg/g) *p* = 0.03 *R* ^2^ = 0.76), (b) overnight changes in nonstructural carbohydrates (mg/g) *p* = 0.03 *R* ^2^ = 0.57, (c) mean annual precipitation (MAP, mm) of each range of origin of each species, *p* = 0.009, *R* ^2^ = 0.63, and (d) Aridity Index, precipitation/potential evapotranspiration of the range of origin of each species, *p* = 0.026, *R* ^2^ = 0.49. Data represent the mean of each species (*n* = 8–10). Plants are defined by water availability (semi‐arid) and temperature (tropical vs. temperate). Triangles represent semi‐arid species, circles represent tropical, and squares represent temperate ecosystem species

The average change in carbohydrate status for NSCs, calculated as the difference between the concentration after rewatering and the concentration during drought, across all R species was positive, indicating that carbohydrate status increased after rewatering (Table 3). In contrast, the average change in carbohydrate status across all NR species was negative (*p* = 0.03; Figure 6). Change in soluble sugar explained more of the variation in XRI_D_ (75%) than total NSCs (56%) and starch (18%).

**Table 3:** Carbohydrates, including total nonstructural carbohydrates (NSC), soluble sugars, and starch, for each species and recovery class

	*Euc ter*, R	*Euc cam*, R	*Aca ite*, R	*Aca cul*, R	*Cas cun*, NR	*Too aus*, NR	*Euc glob*, NR	*Euc sid*, NR
NSC—drought	11.0 (0.3)	12.5 (0.4)	10.7 (0.6)	7.9 (0.2)	10.9 (0.3)	14.8 (0.7)	10.1 (0.5)	12.1 (0.2)
NSC—rewatered	12.3 (0.2)	12.4 (0.1)	11.0 (0.7)	9.8 (0.2)	9.9 (0.4)	11.6 (0.5)	11.2 (0.8)	10.5 (0.5)
Soluble sugars—drought	5.2 (0.5)	6.3 (0.8)	6.9 (0.5)	4.0 (0.4)	5.6 (0.5)	9.2 (0.6)	5.2 (0.7)	6.6 (0.8)
Soluble sugars—rewatered	5.9 (0.5)	6.7 (0.5)	6.5 (0.3)	5.1 (0.4)	4.6 (0.7)	7.0 (0.6)	5.4 (0.5)	5.5 (0.9)
Starch—drought	5.8 (0.3)	6.3 (0.4)	3.7 (0.2)	4.0 (0.2)	5.3 (0.3)	5.5 (0.7)	5.0 (0.5)	5.5 (0.2)
Starch—rewatered	6.4 (0.2)	5.7 (0.1)	4.5 (0.7)	4.7 (0.2)	5.3 (0.4)	4.6 (0.5)	5.8 (0.7)	5.0 (0.5)

Notes

Data represent mean (and standard error in brackets).

NR: class NR, nonrecovering species; R: class R, recovering species.

Species which had higher relative recovery (R species) displayed a positive (8%) accumulation of soluble sugars (and NSCs) on average, while species with lower relative recovery (NR species) experienced a decline of 2%–15% in soluble sugars, supporting a role for solute accumulation in the process of recovery from embolism. The positive correlation between the accumulation of soluble sugars following rewatering relative to drought (Figure 2) suggests a role for sugars in the process of xylem repair. It would seem likely that class R species, with high xylem recovery, were able to increase their soluble sugar content through increased photosynthesis relatively quickly following the alleviation of drought stress. However, our results do not support this hypothesis, given that species with high photosynthesis had a nonsignificant relationship (*p* = 0.12; *R* ^2^ = 0.21) with changes in soluble sugar.

### 3.3 Xylem recovery and nocturnal stomatal conductance

We observed a strong positive correlation (*p* < 0.01; *R* ^2^ = 0.66) between the XRI and nocturnal stomatal conductance (g_s,n_; Figure 5). Increased rates of nocturnal water loss from the canopy via stomatal conductance could increase the water potential gradient, driving the supply of water to refill embolized vessels and provide water to the canopy phloem to transport solutes down the stem to facilitate xylem recovery. Similarly, there were strong correlations between g_s,n_ and changes in soluble sugar and NSC content of stems (Figure 6), further supporting the role of organic solutes in xylem recovery and highlighting the role for nocturnal water movement in xylem recovery.

Xylem repair from embolism is likely to require a continual source of water, both to supply inorganic solutes that may contribute to solute requirements for repair and also to generate turgor gradients and refill xylem vessels (Secchi & Zwieniecki, 2011). Water for refilling may be provided by the phloem, particularly in tall tree species with high canopies and long hydraulic pathways (Brodersen & McElrone, 2013). Both experimental studies (Phillips, Lewis, Logan, & Tissue, 2010; Zeppel et al., 2011) and reviews (Caird et al., 2007; Zeppel et al., 2014) have speculated, but not yet provided evidence for, a role for nocturnal water fluxes in repair of xylem embolism. Our results provide the first such support to our knowledge.

## 4 DISCUSSION

We found evidence for embolism refilling in four of the eight species in our experiment using destructive measurements of PLC under initial well‐watered conditions, during drought, and 12 hr after rewatering, following best practices for these measurements (Klein et al., 2018; Wheeler et al., 2013). Some have recently argued that noninvasive, in vivo observations of embolism repair (i.e., via microtomography) are necessary to demonstrate xylem refilling under tension over short periods (Brodersen & McElrone, 2013. Using these techniques, novel refilling has only been directly observed in grapevine (*Vitis vinifera*; Brodersen et al., 2018), and further, xylem refilling has been observed via sap flow techniques in natural stands of *Populus tremuloides* (Love & Sperry, 2018). Others have argued that when used correctly, destructive techniques for measurement of PLC are a valid method for assessing the ability to recover xylem embolism (Klein et al., 2018; Nardini et al., 2017), and recent research has also demonstrated that repeated microtomography scanning can damage living plant cells and lead to erroneous hydraulic results (Petruzzellis et al., 2018). Note that our data do not allow us to directly test the mechanism of embolism refilling; instead, we examine the physiological traits correlated with the xylem recovery we observed.

### 4.1 Determinants of xylem recovery

Climate of species' biogeographic origin was strongly indicative of recovery. Species that showed xylem recovery were from regions with relatively low rainfall, whereas species with no recovery were from regions with relatively high rainfall (*p* < 0.01, Figure 5). Given that species in drier sites experience water deficits more frequently (or for longer periods) (Zeppel et al., 2015), the evolutionary pressure for embolism repair is likely to be stronger at drier sites than mesic sites. By growing our species in a common and controlled environment, we were able to find strong patterns of biogeographic origin on the prevalence of embolism refilling. Our findings suggest a strong phylogenetic or biogeographic links with the ability to repair emboli. We note, however, that plants were watered to field capacity and embolism refilling occurred when water was abundant.

Plant properties which were linked with xylem recovery included g_s,n_, branch conductivity normalized by sapwood area (*K*_s_; *p* = 0.01, Figure 5c), the ratio of stomatal conductance at night to day, and change in soluble sugars and NSCs (Figure 6a,b). Partial correlation analyses showed that when controlling for the effect of tree size, change in starch, soluble sugars, NSC, and g_s,n_ significantly explained the variation in XRI_D_ (*p* < 0.05). Conversely, xylem recovery was not influenced by photosynthesis, instantaneous water‐use efficiency (WUE, from gas exchange), integrated WUE (from ^13^C analysis), turgor loss point, SLA, or wood density (Table 1). Traits such as P50 and wood density have been suggested as potentially influencing xylem recovery (Ogasa et al., 2013). However, we found that xylem recovery was not significantly influenced by P50 or gas exchange during the day, including photosynthesis or transpiration. Surprisingly, xylem recovery did not vary with hydraulic traits such as turgor loss point, RWC at turgor loss point (RWC_TLP_), osmotic potential at full turgor (_100_) and modulus of elasticity (), relative capacitance at full turgor (C_FT_), and at turgor loss point (C_TLP_) and absolute C_FT_, wood density, stem diameter, and SLA. Linkages between xylem recovery, g_s,n,_ and changes in sugar highlight the interconnections between plant hydraulics and carbon metabolism during drought stress and recovery.

### 4.2 Embolism recovery and time to mortality

Counterintuitively, species with higher xylem recovery of embolism during the rewatering experiment did not have longer times to mortality in the subsequent lethal drought experiment (Figure 5). When controlling for the effect of plant size using partial correlations, the only variable that explained variation in time to death was photosynthetic rate, with species with higher photosynthetic rates dying earlier (*p* = 0.01). Counter to expectations, elevated CO_2_ did not ameliorate mortality or influence XRI. Elevated CO_2_ did decrease stomatal conductance and increase water‐use efficiency, branch conductivity, branch diameter, and leaf temperature. The decreased g_s,d_ and increased water fluxes and tree size appeared to compensate for water savings leading to no effect on xylem recovery or time to mortality. Our results showed that elevated CO_2_ did not significantly increase xylem recovery or prolong time to death. Elevated CO_2_ has been reported to increase nocturnal water fluxes, increase branch conductivity, and alter vulnerability to embolism and xylem anatomy in some, but not all species (Atwell et al., 2007; Domec, Schafer, Oren, Kim, & McCarthy, 2010; Zeppel et al., 2011). This supports the findings of a meta‐analysis of hydraulic properties, which reported a reduced capacity to supply water per unit leaf area, in response to elevated CO_2_ (Mencuccini, 2003).

Droughts may cause tree mortality by inducing embolism blocking the flow of water through plants, leading to hydraulic failure. Accordingly, increasing embolism recovery rates would be expected to prolong time to mortality by maintaining hydraulic conductivity and avoiding desiccation. Counterintuitively, however, time to death did not clearly vary with embolism recovery rates across the eight species in our study. By contrast, higher rates of embolism recovery may have allowed plants to use available water reserves faster, leading to more intense drought stress and reducing the benefits of embolism recovery. Such a strategy could be evolutionarily advantageous in environments where plants compete for a common pool of water, leading to overuse of water (McNickle & Dybzinski, 2013).

### 4.3 Modeling implications

The significant negative relationship between MAP and XRI_D_ (*p* = 0.009) suggests that MAP (or proxies such as biogeographic origin or plant functional types) may be used as a model input to simulate the potential for embolism recovery in vegetation models. Models of the hydraulic continuum that simulate embolism at tree or stand scale are widely available and are actively being incorporated into dynamic global vegetation models. Yet a critical unknown in these models is whether embolism recovery occurs and in which plant species or biomes. Similarly, knowledge of impacts of nocturnal water loss on the water cycle across different biomes is important (Whitley et al., 2013) yet remains scarce in global vegetation models (Lombardozzi et al., 2017). Hydraulic traits have been demonstrated as critical in predicting cross‐species mortality patterns during drought, and cross‐biome comparisons are vital (Adams et al., 2017; Zeppel, 2013), thus simulation both of embolism and embolism recovery will likely be crucial to predicting drought‐induced tree mortality. Our results provide evidence for biome‐specific strategies and reveal that biogeographic origin may be a useful first approach to incorporating embolism recovery in these models.

## 5 CONCLUSIONS

Here, we addressed two fundamental and unresolved issues in plant biology: whether recovery from embolism is influenced by plant water loss at night and identification of traits that influence overnight recovery of embolism. The ability to repair embolism appears to be strongly correlated with MAP for the region of origin for the eight study species. Changes in NSCs were also correlated with the ability to recover from embolism, with larger changes in solute content associated with larger capacity to repair embolisms. Finally, we found that nocturnal stomatal conductance was similarly correlated with differences in embolism recovery, suggesting that nocturnal stomatal conductance is linked with the ability to repair embolism.

Contrary to expectations, we found that the ability to recover from xylem embolism did not delay time to mortality under a subsequent drought. We provide new insight into the trade‐offs in different strategies used by species from different ecosystems, to avoid hydraulic failure and use the transport of carbohydrates to tolerate global‐change‐type droughts. Finally, the link to MAP suggests the importance of evolutionary history and the influence of climate of origin. This has implications for determining plant species tolerance of drought in future climates. In conclusion, we find that biogeographic origin could help predict whether species recover from drought‐induced embolism and ultimately provide modelers the ability to predict recovery from drought in future climates.

## CONFLICT OF INTEREST

None declared.

## AUTHOR CONTRIBUTIONS

M.J.B.Z., W.R.L.A. and H.D.A and S.W.P. designed research; M.J.B.Z., W.R.L.A., P.H., A.C., and D.E. performed research; D.E. and R.R. analyzed stable isotope samples; D.T.T. analyzed leaf carbohydrates; M.J.B.Z, P.H., and A.C. analyzed data; and M.J.B.Z., W.R.L.A., and H.D.A, D.E., A.C., D.T.T., and S.W.P. wrote the paper.

## Data Availability

Data are archived in the publicly accessible repository Figshare, https://doi.org/10.6084/m9.figshare.7807247.
